# Metabolomic Biomarkers for the Detection of Obesity-Driven Endometrial Cancer

**DOI:** 10.3390/cancers13040718

**Published:** 2021-02-10

**Authors:** Kelechi Njoku, Amy E. Campbell, Bethany Geary, Michelle L. MacKintosh, Abigail E. Derbyshire, Sarah J. Kitson, Vanitha N. Sivalingam, Andrew Pierce, Anthony D. Whetton, Emma J. Crosbie

**Affiliations:** 1 Division of Cancer Sciences, Faculty of Biology, Medicine and Health, University of Manchester, 5th Floor Research, St Mary’s Hospital, Oxford Road, Manchester M13 9WL, UK; kelechi.njoku@manchester.ac.uk (K.N.); michelle.mackintosh@mft.nhs.uk (M.L.M.); abiderbyshire@doctors.org.uk (A.E.D.); sarah.kitson@manchester.ac.uk (S.J.K.); vanitha.sivalingam@manchester.ac.uk (V.N.S.); 2 Department of Obstetrics and Gynaecology, Manchester Academic Health Science Centre, Manchester University NHS Foundation Trust, Manchester M13 9WL, UK; 3 Stoller Biomarker Discovery Centre, Division of Cancer Sciences, Faculty of Biology, Medicine and Health, University of Manchester, Manchester M13 9PL, UK; amy.campbell@manchester.ac.uk (A.E.C.); bethany.geary@manchester.ac.uk (B.G.); 4 Wolfson Molecular Imaging Centre, Division of Cancer Sciences, University of Manchester, Palatine Road, Manchester M20 3LJ, UK; andrew.pierce@manchester.ac.uk

**Keywords:** endometrial cancer, obesity, metabolomics, liquid biopsy, mass spectrometry, plasma biomarkers, artificial intelligence

## Abstract

**Simple Summary:**

Endometrial cancer is the commonest cancer of the female genital tract and obesity is its main modifiable risk factor. Over 80% of endometrial cancers develop in the context of obesity-induced metabolic changes. This study focuses on the potential of plasma-based metabolites to enable the early detection of endometrial cancer in a cohort of women with body mass index (BMI) ≥ 30 kg/m^2^. Specific lipid metabolites including phospholipids and sphingolipids (sphingomyelins) demonstrated good accuracy for the detection of endometrial cancer, especially when combined in a diagnostic model. This study advances our knowledge of the role of metabolomics in endometrial cancer and provides a basis for the minimally invasive screening of women with elevated BMI.

**Abstract:**

Endometrial cancer is the most common malignancy of the female genital tract and a major cause of morbidity and mortality in women. Early detection is key to ensuring good outcomes but a lack of minimally invasive screening tools is a significant barrier. Most endometrial cancers are obesity-driven and develop in the context of severe metabolomic dysfunction. Blood-derived metabolites may therefore provide clinically relevant biomarkers for endometrial cancer detection. In this study, we analysed plasma samples of women with body mass index (BMI) ≥ 30 kg/m^2^ and endometrioid endometrial cancer (cases, *n* = 67) or histologically normal endometrium (controls, *n* = 69), using a mass spectrometry-based metabolomics approach. Eighty percent of the samples were randomly selected to serve as a training set and the remaining 20% were used to qualify test performance. Robust predictive models (AUC > 0.9) for endometrial cancer detection based on artificial intelligence algorithms were developed and validated. Phospholipids were of significance as biomarkers of endometrial cancer, with sphingolipids (sphingomyelins) discriminatory in post-menopausal women. An algorithm combining the top ten performing metabolites showed 92.6% prediction accuracy (AUC of 0.95) for endometrial cancer detection. These results suggest that a simple blood test could enable the early detection of endometrial cancer and provide the basis for a minimally invasive screening tool for women with a BMI ≥ 30 kg/m^2^.

## 1. Introduction

Endometrial cancer is the most common gynaecological malignancy in the United Kingdom, where its incidence is rising in parallel with the obesity epidemic [1]. Obesity is the major risk factor for type I cancers of low-grade endometrioid morphology, with every 5 kg/m^2^ increase in body mass index (BMI) linked to a 60% increased cancer risk [2]. Almost half of all endometrial cancers are attributed to overweight (BMI ≥ 25 kg/m^2^) and obesity (BMI ≥ 30 kg/m^2^) [3]. The strong dose–response relationship portends a 10–15% lifetime risk of endometrial cancer in women with class III obesity (BMI ≥ 40 kg/m^2^) compared with a population average of 2% [4]. Whilst its aetiological importance is clear, the biology underpinning obesity-driven endometrial carcinogenesis is incompletely understood [5]. Adipose tissue is a rich source of oestrogens that stimulate endometrial proliferation, particularly when unopposed by progesterone in postmenopausal and anovulatory states [6]. Metabolically unhealthy obesity, rather than excess bodyweight per se, is of particular aetiological significance, with impaired glucose tolerance and chronic insulin resistance acting synergistically to increase endometrial cancer risk [7]. Type 2 diabetes mellitus is associated with a 62% upsurge [8], and uncontrolled diabetes mellitus a nearly five-fold greater susceptibility to endometrial cancer [9]. 

A recent study found occult endometrial abnormalities in 14% of women with class III obesity referred for weight loss management [10]. All but one had low-grade early-stage endometrial cancer or its precursor lesion, atypical hyperplasia. The early identification of these abnormalities in asymptomatic women could enable conservative management strategies that preserve fertility and/or reduce the morbidity of surgery [11,12]. Yet, no current screening programme exists for these high-risk women, partly because current diagnostics are invasive with low acceptability profiles and/or poor diagnostic accuracy [13]. A simple, minimally invasive endometrial cancer screening tool that can triage high-risk women for diagnostic workup, whilst safely reassuring those at low risk, would represent a major advance in the field [14,15]. 

High-throughput technologies and machine learning techniques have emerged as powerful tools for biomarker discovery and validation [15,16,17,18,19]. Metabolomics studies the downstream products of genomic, transcriptomic, and proteomic processes and best mirrors the human phenotype [20,21]. Thus, metabolomics has great potential to deliver clinically relevant biomarkers for endometrial cancer detection [22]. A blood-based test for cancer has broad appeal, being rated the second most important research priority for detecting cancer early in our recent James Lind Alliance Priority Setting Partnership [23]. A significant challenge is identifying cancer-relevant biomarkers within the context of severe metabolic dysfunction that characterises endometrial cancer risk. Here, we investigate the potential of plasma-based metabolites to detect endometrial cancer in a cohort of women with class III obesity, using a mass spectrometry-based metabolomics approach. 

## 2. Materials and Methods

### 2.1. Study Population

This study included women with BMI ≥ 30 kg/m^2^ participating in clinical research, who donated blood samples and gave written, informed consent for their pseudo-anonymised data to be used for future research. The primary research studies received approval from the North West and Cambridge East Research Ethics Committees and were conducted according to the principles of the Declaration of Helsinki. Cases and controls were recruited at Manchester University and Salford Royal NHS Foundation Trusts, United Kingdom. Cases were confirmed to have endometrioid endometrial cancer based on specialist histopathological assessment of biopsy and/or hysterectomy specimens [24,25]. Controls were women referred for weight loss management and confirmed to have normal histology on endometrial biopsy [10]. Clinicopathological data included age, BMI, smoking status, menopausal status, parity, type 2 diabetes mellitus status and medications used. All tissue specimens were assessed by at least two specialist gynaecological pathologists reporting according to UK Royal College of Pathology standards. Blood samples were collected following an overnight fast. Study investigators were blinded to the clinical information and biopsy results of subjects during acquisition of metabolomics data.

### 2.2. Metabolomic Profiling

Blood samples were collected in standard EDTA tubes, centrifuged at 2000 rpm for 10 min and the supernatant (plasma) was collected and stored at −80 °C. The samples were subsequently shipped to Metabolon Inc^®^, Durham, NC, USA, on dry ice and maintained at −80 °C until processed. Non-targeted MS metabolomic analysis was performed by Metabolon Inc^®^, according to company protocols and is summarised below.

#### 2.2.1. Sample Preparation 

Sample preparation was carried out using the automated MicroLab STAR^®^ liquid handling system (Hamilton Company, Reno, NV, USA). Recovery standards were added to the samples prior to extraction for quality control purposes. To optimise the recovery of chemically diverse metabolites, proteins were removed by precipitation with methanol under vigorous shaking GenoGrinder 2000 by Glen Mills Inc., Clifton, NJ, USA) followed by centrifugation. The resulting extract was split into four aliquots and prepared for subsequent analysis using solvents compatible with the various separation and detection methods. Zymark TurboVap concentration evaporator (SOTAX AG, Aesch, Switzerland) was used to remove organic solvents. 

#### 2.2.2. Metabolite Separation and Detection

Multiple methods were used for metabolite separation and identification to maximise the number of metabolites detected. All methods were performed using a Waters ACQUITY ultra-performance liquid chromatography (UPLC) system (Waters Corporation, Milford, MA, USA) and a Thermo Scientific Q-Exactive high resolution/accurate mass spectrometer (ThermoFisher Scientific, Waltham, MA, USA). This was interfaced with a heated electrospray ionisation (HESI-II) source and Orbitrap mass analyzer operating at 35,000 mass resolution. Three sample extract aliquots were analysed using reversed phase UPLC with tandem mass spectrometry (RP UHPLCMS/MS). A positive ion mode electrospray ionisation (ESI) was used for two aliquots chromatographically optimised for more hydrophilic and more hydrophobic compounds, respectively, and a negative ion mode ESI for the third aliquot. The fourth aliquot was analysed using negative ion mode ESI following elution from a hydrophilic interaction liquid chromatography column (HILIC UPLCMS/MS). The chromatographic conditions used and optimised for the various metabolite species are summarised in Table S1.

#### 2.2.3. Metabolite Identification 

Raw data including molecular and fragment ions were searched against a reference library of over 14,000 metabolites based on authenticated standards. Metabolites were identified based on their chromatographic features (including MS/MS spectra), retention time/index (RI) and mass-to-charge ratio (*m/z*). The specific criteria used for biochemical identification included a retention index within a narrow window of the proposed identification and an accurate mass match to the library ± 10 ppm. MS/MS forward and reverse scores were used to control for false discovery rates. Ions that lacked a definite biochemical identity were given a numerical designation. Data curation was carried out by Metabolon, Inc, Durham, NC, USA data analysts to ensure accurate and consistent identification of metabolites as well as removal of artefacts, misassignments and background noise. Peak quantification was carried out using area under the curve analysis. Comparison of the peak area of a given metabolite in the sample to the peak area of a standard of known concentration was used to determine the metabolite concentration. 

#### 2.2.4. Data Pre-Processing 

Metabolite concentrations were reported in the form of standardised intensities. Each metabolite concentration was rescaled to set the median equal to 1 (by dividing the concentration of each metabolite by the median). Thus, the concentration of a given metabolite in a given sample was made relative to the median concentration of all the samples processed as part of the study. The presence of missing values in this study was indicated by the concentration of a given metabolite falling below an assay’s limit of detection (LOD). Missing metabolite concentrations were imputed with a standardised intensity set at the minimum detected value for that compound.

### 2.3. Data Analysis 

All statistical analyses were performed using R version 3.2.5 (R Development Core Team, Vienna, Austria), STATA version 16, and MetaboAnalyst 4.0. The Shapiro–Wilk test was used to assess normality of continuous variables. Descriptive analyses of the study demographic data (continuous and categorical) were performed using means (±standard deviations) and counts (%), respectively, with differences between groups assessed using Student’s *t*-test for continuous variables and the chi-square test for categorical variables. The majority of the metabolite concentrations (median scaled standardised intensity) were not normally distributed. As such, non-parametric tests were used in subsequent analysis. Specifically, the Mann–Whitney *U* test was used to compare metabolite concentrations in the cancer group versus control group and for other group comparisons made. We applied a false discovery rate adjustment for multiple testing using the Benjamini–Hochberg correction method (q = 0.05). A computation of the ratio of metabolite concentrations in cases and controls was used to identify the direction and degree of fold change and allowed for the identification of the groups of metabolites with unidirectional alterations. Principal component analysis (PCA) and t-distributed stochastic neighbour embedding (t-SNE) plots were used to assess degree of separation between groups. Random forest modelling was used to identify the best-performing biomarkers and to develop predictive models for the detection of endometrial cancer. Eighty per cent of the samples were randomly selected to serve as a “training set” and the remaining 20% were used to test the model. Heat maps were generated based on hierarchical clustering of the top discriminatory metabolites using the Euclidean distance measure and the Ward algorithm. Row scaling (heat maps) was performed for each metabolite by the subtraction of the mean from each feature and then dividing by the standard deviation. Area under the receiver-operator characteristic curves (AUC) and the 95% confidence intervals were computed for both metabolites and metabolomics signatures. The selection of cut-off points was based on the Youden Index (J = max {Sensitivity + Specificity − 1}).

An overview of the study workflow is summarised in Figure S1.

## 3. Results

### 3.1. Participant Demographics

The study comprised 136 women with BMI ≥ 30kg/m^2^ of whom 67 had endometrioid endometrial cancer (cases) and 69 had histologically normal endometrium (controls). The median age and BMI for the cohort was 54 years (IQR 43, 65) and 46 kg/m^2^ (IQR 39, 52) respectively. Cases were older and more likely to be post-menopausal and nulliparous while controls were more obese. The majority of the endometrial cancers were low-grade (91.0% grades I/II), early-stage (88.0% stage I) cancers with lymphovascular space invasion occurring in only 12 women (18.0% of cases) (Table 1). Participant demographics and clinicopathological characteristics are summarised in Table 1.

### 3.2. Metabolomic Analysis of Plasma Samples

**Figure 1:**
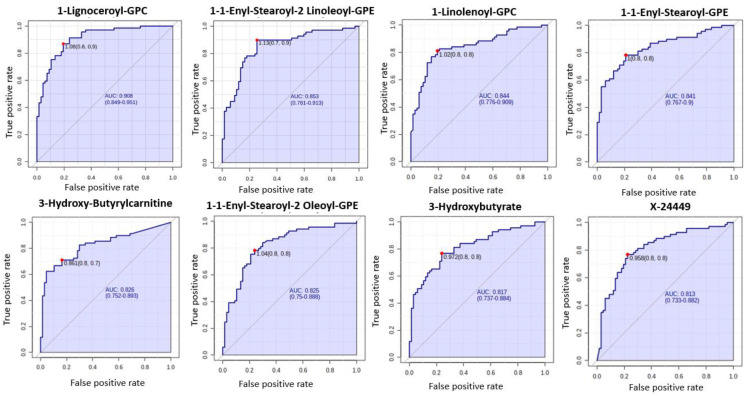
Receiver operating characteristic (ROC) curves of the promising endometrial cancer diagnostic biomarkers from different classes based on the area under the curve (AUC) analyses of *n* = 67 cancers and *n* = 69 controls. The optimal cut-off was based on the closest to the top left corner principle and is indicated by the red dot in the ROC curves. Metabolites starting with X are unnamed; the pathways of these are unknown. GPC—Glycerophosphocholine. GPE—Glycerophosphoethanolamine.

**Figure 2:**
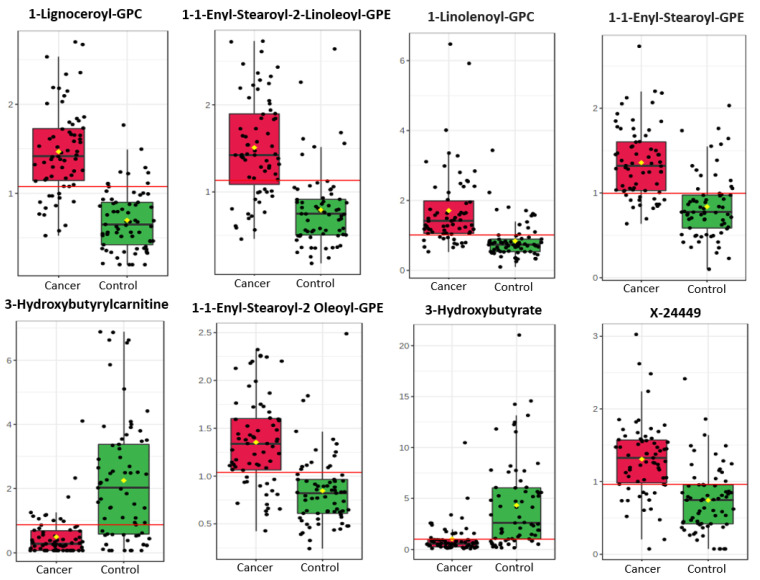
Box plot distribution of promising endometrial cancer diagnostic metabolites based on analyses of *n* = 67 cancers and *n* = 69 controls. The black dots along the Y axis in the box plots represent the concentrations of each metabolite while the yellow diamond represents the mean concentration for the group. The notch represents the 95% confidence interval around the median of each group. The horizontal red lines represent the optimal cut-off. Metabolites starting with X are unnamed; the pathways of these are unknown.

**Figure 3:**
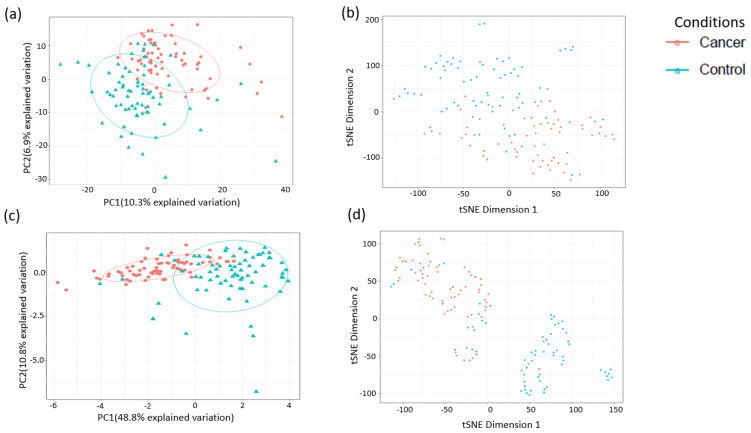
Analysis of sample separation using the training set (*n* = 109, cancers = 54, controls = 55) based on principal component (PCA) (**a**,**c**) and t-distributed stochastic neighbour embedding (t-SNE) (**b**,**d**) analyses using all identified metabolites (**a**,**b**) and the top 10 discriminatory metabolites (**c**,**d**) identified by random forest machine learning technique. t-SNE (perplexity: 5, iteration: 10,000).

**Figure 4:**
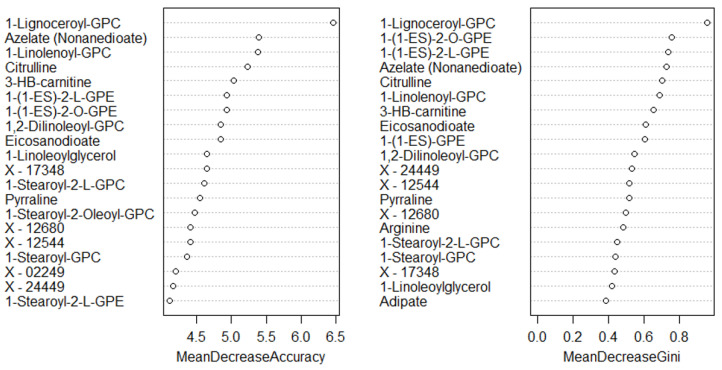
Top 20 discriminatory metabolites identified by random forest machine learning technique and ranked by their contribution to classification accuracy using mean decrease accuracy and mean decrease gini index (node impurity) based on the training set (*n* = 109, cancers = 54, controls = 55). Metabolites starting with X are unnamed; the pathways of these are unknown.

**Figure 5:**
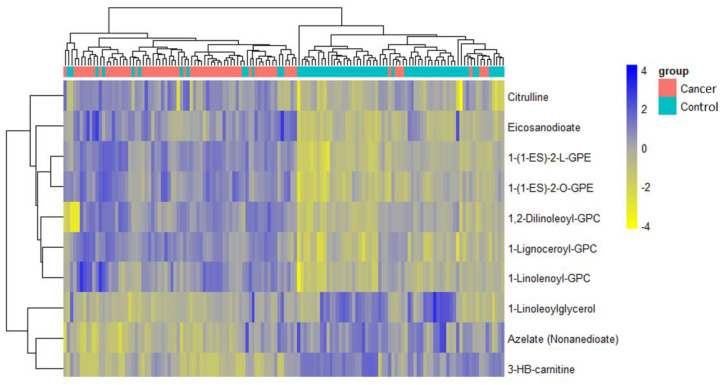
Hierarchical clustering using the top 10 discriminatory metabolites in the training set (*n* = 109, cancers = 54, controls = 55) based on mean decreasing accuracy. The difference in intensities of the top 10 metabolites by cancer-control status is shown. Each coloured cell in the map represents scaled/relative concentration of indicated metabolite. Metabolites are clustered along the vertical axis while subjects are clustered along the horizontal axis. Hierarchical clustering was based on the Euclidean distance measure and the Ward algorithm.

A total of 1137 metabolites were quantified in the study plasma samples of which 733 (64.5%) were biochemically defined. These included amino acids, fatty acids, biogenic amines, sphingolipids, steroids, hexoses, nucleotides, phospholipids, vitamins and xenobiotics. The remaining 35.5% were unnamed biochemical entities, the pathways of which are unknown. We performed classical univariate ROC curve analyses of individual biomarkers to identify putative biomarkers for the discrimination of endometrial cancer from controls (Figure 1). In this analysis, 1-Lignoceroyl GPC (24:0), 1-(1-enyl-stearoyl)-2-linoleoyl-GPE (P-18:0/18:2) and 1-linolenoyl-GPC (18:3) were the most discriminatory biomarkers with AUCs of 0.91 (95%CI 0.86–0.95), 0.85 (95%CI 0.78–0.91) and 0.84 (95% CI 0.78–0.91), respectively. Phosphatidylcholines (PCs) thus feature as potentially important biomarkers. Other discriminatory biomarkers included 3-hydroxylbyryl carnitine and 3-hydroxybutyrate with AUCs of 0.83 and 0.82, respectively (see Figure 1 and Figure 2). Principal component analysis (PCA) and t-distributed stochastic neighbour embedding (t-SNE) were employed and showed some discrimination between cancers and controls (Figure 3a,b). Random forest machine learning was then applied and identified the top 20 discriminatory biomarkers. These were ranked by their contributions to the classification accuracy based on the mean decrease accuracy metric and the mean decrease gini index (Figure 4). A PCA and t-SNE plot based on the top ten discriminatory biomarkers showed a strong degree of separation between cancers and controls (Figure 3c,d). Hierarchical clustering was subsequently performed based on the top 10 discriminatory biomarkers and a heat map was generated (Figure 5). The random forest algorithm was used to split the samples 80:20, 80% for the training set and 20% for testing. The algorithm demonstrated an accuracy of 86.2% (OOB error rate of 13.76%) in the training set, 92.6% prediction accuracy in the testing set and an AUC of 0.95 for endometrial cancer detection (Table 2 and Table 3). Biochemical identities, super-pathways and sub-pathways of discriminatory metabolites for EC detection are summarized in Table S2. ROC curves based on the Random Forest diagnostic algorithms are shown in Figure S2.

### 3.3. Metabolomic Analysis for the Detection of Early-Stage Endometrial Cancer

**Figure 6:**
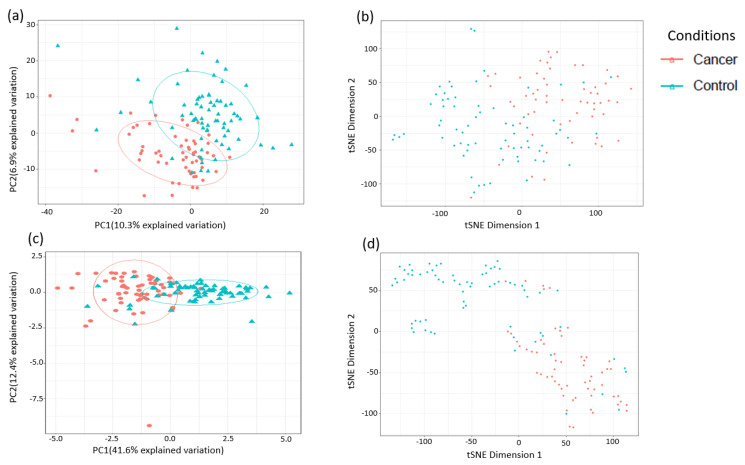
Analysis of sample separation (comparing early-stage (stage 1) endometrial cancer versus controls (*n* = 102, cancers = 47, controls = 55) based on PCA (**a**,**c**) and t-distributed stochastic neighbour embedding (t-SNE) (**b**,**d**) analyses using all identified metabolites (**a**,**b**) and the top 10 discriminatory metabolites (**c**,**d**) identified by random forest machine learning technique. t-SNE (perplexity: 5, iteration: 10,000).

**Figure 7:**
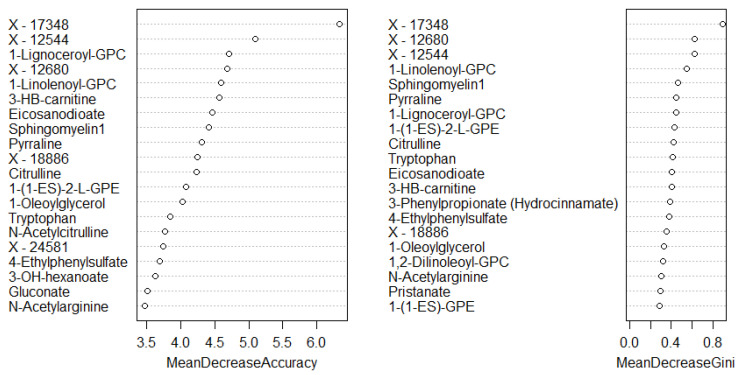
Top 20 discriminatory metabolites for the detection of early-stage endometrial cancer based on the training set (*n* = 102, cancers = 47, controls = 55) identified by random forest machine learning technique and ranked by their contribution to classification accuracy using mean decrease accuracy and mean decrease gini index. Metabolites starting with X are unnamed; the pathways of these are unknown.

**Figure 8:**
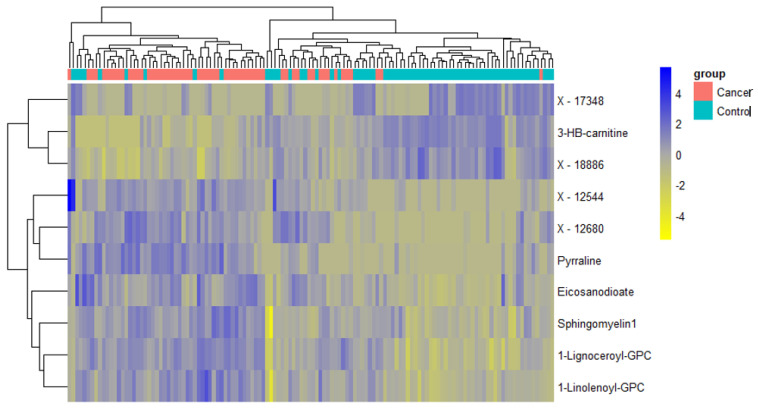
Hierarchical clustering using the top 10 discriminatory metabolites for the detection of early-stage endometrial cancer in the training set (*n* = 102, cancers = 47, controls = 55) based on mean decreasing accuracy using random forest classification algorithm. The difference in intensities of the top 10 metabolites by cancer-control status is shown. Each coloured cell in the map represents scaled/relative concentration of indicated metabolite. Metabolites are clustered along the vertical axis while subjects are clustered along the horizontal axis. Metabolites starting with X are unnamed; the pathways of these are unknown.

It is important that plasma metabolites used for the identification of endometrial cancer can detect early-stage, not just advanced-stage, disease. We therefore sought to identify metabolites able to distinguish stage 1 endometrial cancer (*n* = 59) from controls (*n* = 69). PCA and t-SNE analyses showed good discrimination between stage 1 disease and controls on all study metabolites (Figure 6a,b) and based on the top 10 metabolites identified using random forest modelling (Figure 6c,d). The top 20 metabolites that distinguished stage 1 endometrial cancer from controls based on random forest algorithm are summarised in Figure 7 and their contribution to the classification accuracy ranked by the mean decrease accuracy and mean decrease gini index. Glycerophospholipids remained important predictors of stage 1 disease, however, the top discriminatory metabolites were uncharacterised chemical entities. Hierarchical clustering using the top 10 metabolites was performed and the generated heat map presented in Figure 8. This showed good discrimination between stage 1 endometrial cancer and controls based on selected metabolites. The study samples were subsequently split 80:20 (80% training set and 20% testing set) using random forest algorithm. The diagnostic algorithm demonstrated an OOB error rate of 14.7% in the training set, a prediction accuracy of 84.6% in the testing set and an AUC of 0.98 for stage 1 endometrial cancer detection (Table 4 and Table 5).

### 3.4. Metabolomic Biomarkers for Predicting Deep Myometrial Invasion and LVSI

**Figure 9:**
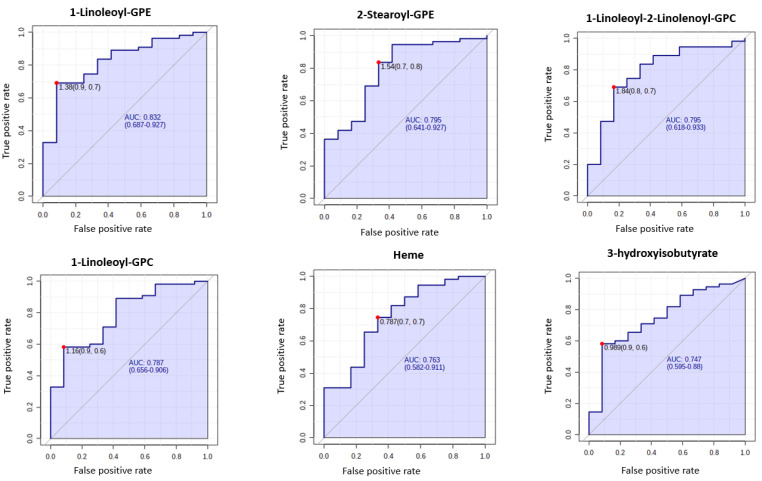
ROC curves of the promising biomarkers for the prediction of lymphovascular space invasion (*n* = 12) based on AUC analyses of *n* = 67 cancers. The optimal cut-off was based on the closest to the top left corner principle and is indicated by the red dot in the ROC curves. Metabolites starting with X are unnamed; the pathways of these are unknown.

**Figure 10:**
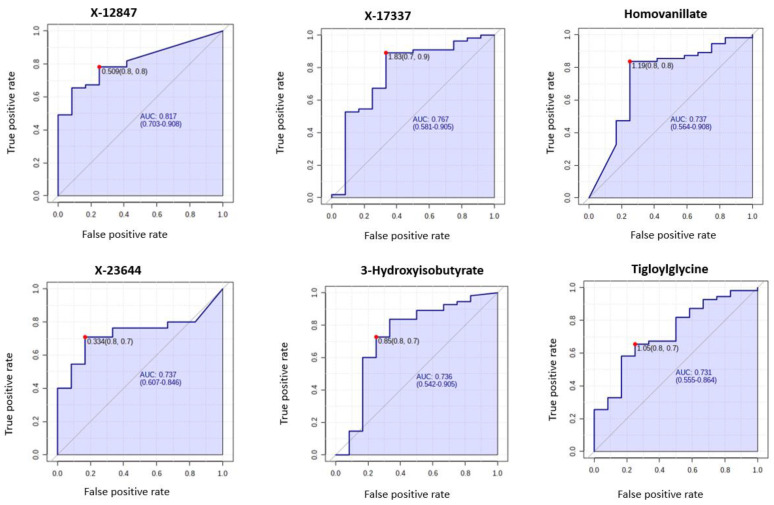
ROC curves of the promising biomarkers for the prediction of deep myometrial invasion (*n* = 12) based on AUC analyses of *n* = 67 cancers. The optimal cut-off was based on the closest to the top left corner principle and is indicated by the red dot in the ROC curves. Metabolites starting with X are unnamed; the pathways of these are unknown.

Lymphovascular space invasion (LVSI) and deep myometrial invasion are important endometrial cancer prognostic biomarkers. However, their characterisation in clinical practice is performed by histopathologists with moderate interobserver reproducibility. Metabolites with the potential to predict deep myometrial invasion and LVSI will significantly improve endometrial cancer prognostic characterisation. We therefore sought to identify metabolites that can predict LVSI (*n* = 12) and deep myometrial invasion (*n* = 12) in women with endometrioid endometrial cancer. We limited our analysis to univariate ROC curve analysis and identified specific glycerophosphoethanolamines, glycerophosphocholines, heme and hydroxybutyrate as important predictors of LVSI with AUCs ranging from 0.75–0.83 (Figure 9). A number of unnamed metabolites were noted to predict deep myometrial invasion in addition to Homovanillate, 3-OH-isobutyrate and Tigloylglycine with AUCs ranging between 0.73 and 0.82 (Figure 10). 

### 3.5. Consideration of Potential Confounding Factors

**Figure 11:**
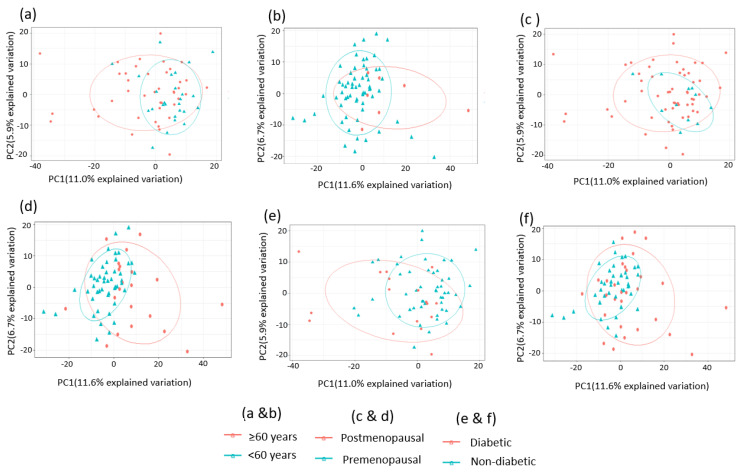
Score plots generated after unsupervised PCA to visualise differences and similarities according to confounding factors. (**a**,**b**) Score plots according to age (<60 years; ≥60 years) for cancers (**a**) and controls (**b**). (**c**,**d**) Score plots according to menopausal status for cancers (**c**) and controls (**d**). (**e**,**f**) Score plots according to diabetes (present; not present) for cancers (**e**) and controls (**f**).

In order to confirm that the discriminatory power of the metabolite signature was due to the presence and absence of endometrial cancer and not confounding variables, we carried out further analyses, taking into consideration the effects of age, BMI, menopausal and diabetic status. First, we performed unsupervised exploratory analyses using score plots generated from PCAs to identify differences between groups (Figure 11). The PCA score plots showed a mild segregation pattern in the confounding factor comparisons suggesting that age, menopausal and diabetic status could potentially have influenced the diagnostic performance within groups of samples (Figure 11). However, these analyses were limited by small numbers within groups. Next, we performed pairwise Spearman’s correlation analysis with Bonferroni correction looking at the correlation between age, BMI and selected metabolites (Table 6). There was no evidence of a strong correlation between the metabolite concentrations and age, BMI or parity. Correlation coefficients ranged between 0.25–0.45 for age-based comparisons, 0.33–0.58 for BMI-based comparisons and 0.21–0.32 for parity-based comparisons, suggesting weak correlations between age, BMI, parity and selected metabolite concentrations. While the glycerophospholipids (GPC, GPE) had a positive correlation with age and a negative correlation with BMI/parity, the reverse was the case for the hydroxybutyrates. 

**Figure 12:**
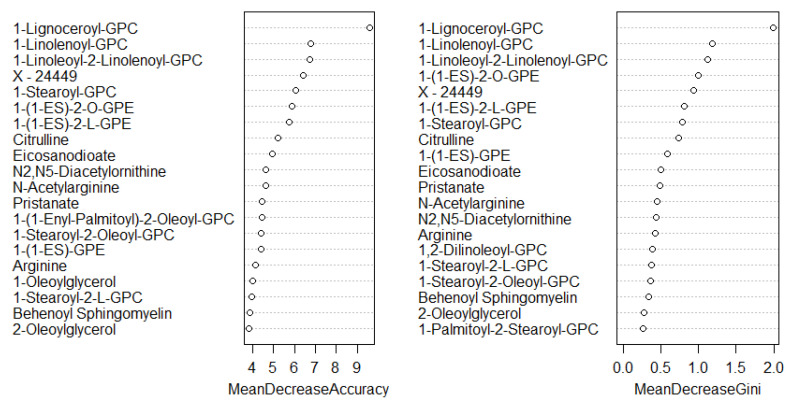
Top 20 discriminatory metabolites for the detection of endometrial cancer following exclusion of women with type 2 diabetes mellitus (training set: *n* = 72, cancers = 40, controls = 32) Metabolites were identified by random forest machine learning technique and ranked by their contribution to classification accuracy using mean decrease accuracy and mean decrease gini index. Metabolites starting with X are unnamed; the pathways of these are unknown.

**Figure 13:**
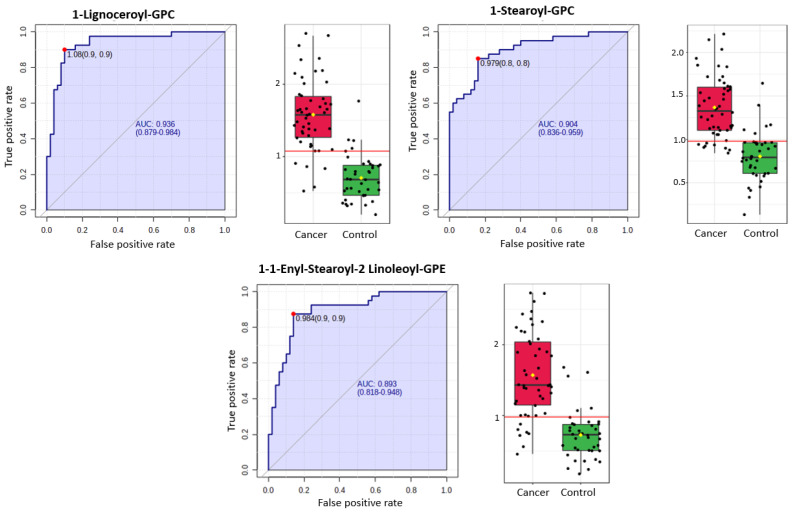
ROC curves of selected metabolites for endometrial cancer detection after exclusion of women with type 2 diabetes mellitus (*n* = 90, cases = 50, controls = 40) based on AUC analysis. The optimal cut-off was based on the closest to the top left corner principle and is indicated by the red dot in the ROC curves.

**Figure 14:**
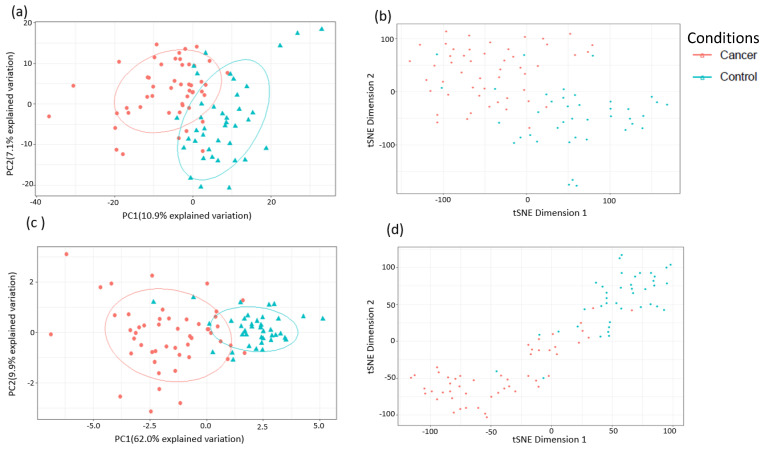
Analysis of sample separation after exclusion of women with type 2 diabetes mellitus (training set: *n* = 72, cancers = 40, controls = 32) based on PCA (**a**,**c**) and t-distributed stochastic neighbour embedding (t-SNE) (**b**,**d**) analyses using all identified metabolites (**a**,**b**) and the top 10 discriminatory metabolites (**c**,**d**) identified by random forest machine learning. t-SNE (perplexity: 5, iteration: 10,000).

**Figure 15:**
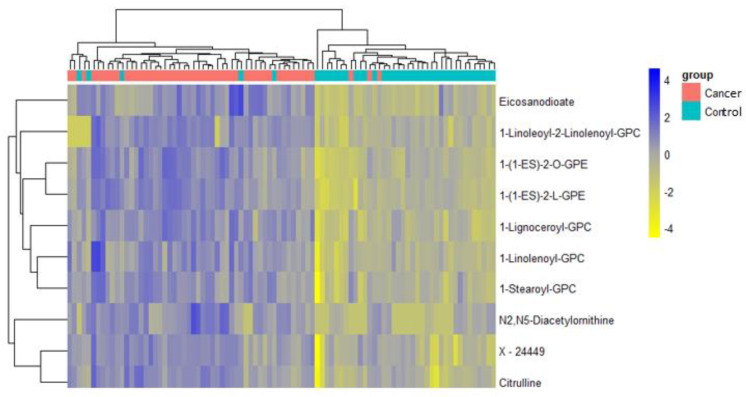
Hierarchical clustering using the top 10 discriminatory metabolites for the detection of endometrial cancer after exclusion of women with type 2 diabetes mellitus (training set: *n* = 72, cancers = 40, controls = 32). Discriminatory metabolites were based on mean decreasing accuracy metric from random forest analysis. The difference in intensities of the top 10 metabolites by cancer-control status is shown. Each coloured cell in the map represents the scaled/relative concentration of indicated metabolite. Metabolites are clustered along the vertical axis and subjects along the horizontal axis. Metabolites starting with X are unnamed with unknown pathways.

We then applied an exclusion principle by eliminating women with type 2 diabetes mellitus, leaving 50 cancers and 40 controls. There was still a difference between cases and controls by menopausal status. The list of the top-performing metabolites remained largely similar (Figure 12) based on our machine learning (ML) approaches, suggesting that diabetic status did not significantly affect the diagnostic performance of the metabolites. A receiver characteristics curve analysis of these metabolites gave an AUC of 0.94, 0.90 and 0.89 for 1-Lignoceroyl GPC, 1-Steroyl GPC and 1-1 Enyl-Steroyl-2-Linoleoyl-GPE, respectively (Figure 13). The PCA analyses and heat maps also showed good discrimination between cancer cases and controls (Figure 14 and Figure 15), confirming that diabetes status was not a significant confounder in the study analyses, especially with respect to the diagnostic performance of the glycerophospholipids. However, we noted that the hydroxybutyrates and their derivatives were no longer important discriminators of cancers from controls following exclusion of women with type 2 diabetes mellitus (Figure 12), suggesting that their diagnostic ability may be related to their association with diabetes mellitus. The samples of women with no clinical or biochemical evidence of diabetes mellitus were split 80:20 (80% training set and 20% testing set) with the training data used to build a model to separate cancers from controls. The random forest model had an OOB error rate of 11.1% and when tested using the remaining 20% data, it gave a prediction accuracy of 88.9% (Table 7 and Table 8).

**Figure 16:**
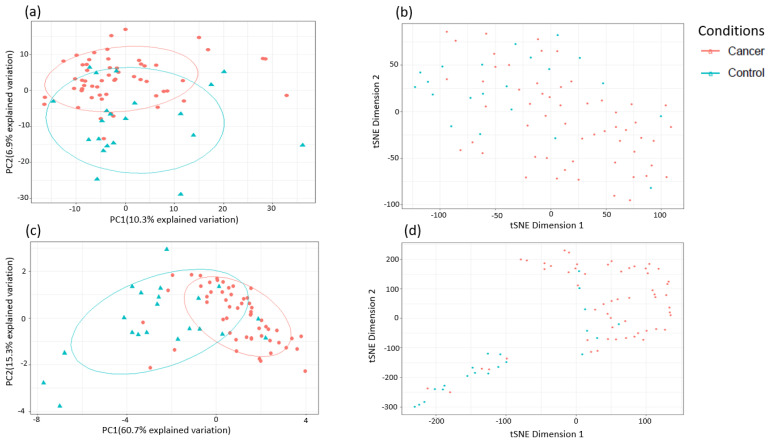
Analysis of sample separation for post-menopausal women (*n* = 77, cases = 56, controls = 21) based on PCA (**a**,**c**) and t-distributed stochastic neighbour embedding (t-SNE) (**b**,**d**) analyses using all identified metabolites (**a**,**b**) and the top 10 discriminatory metabolites (**c**,**d**) identified by random forest machine learning. t-SNE (perplexity: 5, iteration: 10,000).

**Figure 17:**
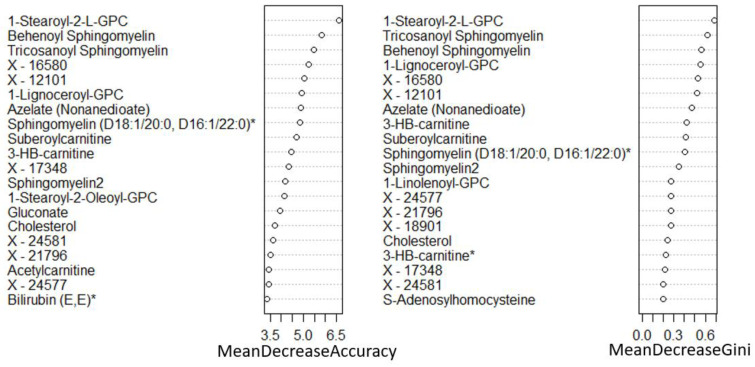
Top 20 discriminatory metabolites for the detection of endometrial cancer in post-menopausal women (*n* = 77, cases = 56, controls = 21). Metabolites were identified by random forest machine learning and ranked by their contribution to classification accuracy using mean decrease accuracy metric and mean decrease gini index. Metabolites starting with X are unnamed; the pathways of these are unknown.

**Figure 18:**
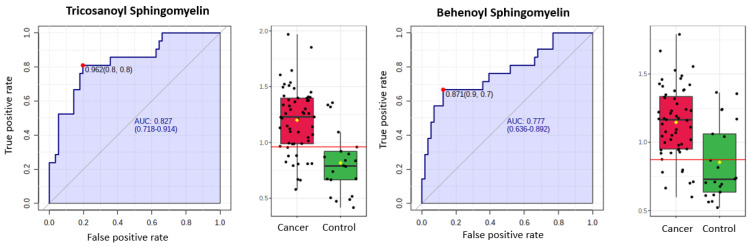
ROC and box-plot distributions of selected metabolites (sphingomyelins) for endometrial cancer detection in post-menopausal women (*n* = 77, cases = 56, controls = 21) based on AUC analysis. The optimal cut-off was based on the closest to the top left corner principle and is indicated by the red dot in the ROC figures. The black dots in the box plots represent the concentrations of each metabolite, while the red diamond represents the mean concentration for the group. The notch represents 95% confidence interval around the median of each group.

**Figure 19:**
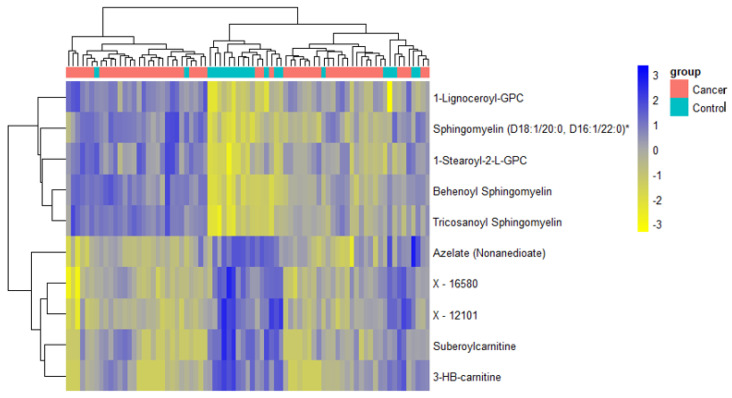
Hierarchical clustering using the top 10 discriminatory metabolites for the detection of endometrial cancer in post-menopausal women (*n* = 77, cases = 56, controls = 21). Discriminatory metabolites were based on mean decrease accuracy metric using random forest analysis. The difference in intensities of the top 10 metabolites by cancer-control status is shown. Each coloured cell in the map represents the scaled concentration of indicated metabolite. Metabolites are clustered along the vertical axis while subjects are clustered along the horizontal axis. Metabolites starting with X are unnamed; the pathways of these are unknown.

Finally, we restricted the analysis to post-menopausal women (*n* = 77, cases = 56, controls = 21). There was still a difference according to diabetes status between cancers and controls in this cohort (*p* = 0.001). The PCA and t-SNE plots showed good discrimination between cancers and controls based on all study metabolites and on the top 10 discriminatory metabolites (Figure 16). The glycerophospholipids remained important predictors of endometrial cancer. The 3-hydroxybutyrate derivatives were also important predictors of endometrial cancer (ranked in the top 10 based on random forest mean decrease accuracy and mean decrease gini index) (Figure 17), confirming their likely association with type 2 diabetes mellitus. Importantly, we noticed the sphingolipids, specifically sphingomyelins, to be well represented in the top 10 discriminatory biomarkers in post-menopausal women (Figure 17). Tricosanoyl and Behenoyl sphingomyelins, in particular, demonstrated AUCs of 0.83 and 0.78, respectively (Figure 18). Hierarchical clustering also showed good discrimination based on the top 10 metabolites in this cohort (Figure 19). 

## 4. Discussion 

In this study, we evaluated the potential of plasma-based metabolomic biomarkers to detect endometrial cancer in women with class III obesity. Top-performing metabolites, particularly glycerophospholipids and hydroxybutyrates, showed good accuracy for endometrial cancer detection, with AUCs > 0.80. An algorithm combining the ten most discriminatory metabolites was even more successful, with AUCs > 0.90. Potential sources of confounding, particularly age, BMI and diabetes status, did not demonstrate strong correlations with individual metabolites, with the exception of hydroxybutyrates and type 2 diabetes mellitus. These data suggest that a simple blood test could offer a minimally invasive endometrial cancer detection tool for women with class III obesity.

The rising prevalence of endometrial cancer has stimulated an interest in biomarker discovery alongside minimally invasive sampling technologies for its early detection [11]. Many studies have explored the possibility of detecting endometrial cancer in blood using genetic biomarkers (including tumour DNA [26], epigenetic modifications [27] and transcripts [28,29]), proteins [18,30] and metabolites [19,22] through genomic, epigenomic, transcriptomic, proteomic, spectroscopic and metabolomic approaches. The metabolome reflects the functional human phenotype and as such, has enormous potential to deliver clinically relevant biomarkers for endometrial cancer detection [20,31]. Indeed, metabolic reprogramming is a defining hallmark of carcinogenesis [32]. Pertubations in critical pathways involving fatty acid metabolism, choline metabolism, tricarboxylic acid cycle and glycolysis have all been described in the pathogenesis of cancer [21,33,34]. Metabolomic biomarkers have shown promise for the early detection of several cancers, including those of the breast [35], colon [36] and prostate [37], and may be particularly relevant in endometrial cancer, given its strong association with obesity, insulin resistance and type 2 diabetes mellitus [38].

Our finding that glycerophospholipids are important diagnostic biomarkers in endometrial cancer is consistent with published data [39,40,41,42]. Glycerophospholipids are the main components of biological membranes and, alongside fatty acids, glycerolipids, sphingolipids and sterols, have been linked to cancer development [43]. The upregulation of phospholipid biosynthetic pathways in cancer cells is a direct consequence of accelerated growth and enhanced membrane biosynthesis that accompanies tumorigenesis [44]. A recent systematic review by our group identified choline derivatives, specifically glycerophosphocholines and phosphocholines, as promising biomarkers for endometrial cancer detection [22]. Altered choline metabolism is a hallmark of carcinogenesis and is linked to mitogenic signal transduction, the regulatory mechanism that modulates cell proliferation, differentiation, metabolism and death [34,45,46]. Up-regulation of choline-containing precursors, including phosphocholines and total choline-containing compounds, is caused by the overexpression and activation of several key enzymes involved in choline metabolism by cancer cells. These processes are mediated by oncogenic signalling pathways, including RAS and PI3K-AKT [46,47]. Trousil and colleagues found that altered choline metabolism in endometrial cancer is caused by an overexpression of choline kinase alpha and hyperactivation of the deacylation pathway [48]. Choline derivatives are detectable in blood, tumour and vaginal fluid in women with endometrial cancer [39,40,41]. They have also been described in breast, prostate and other solid tumours [46]. 3-hydroxybutyrate and its derivatives have also shown promise for endometrial cancer detection [49,50]. Bahado-Singh found that 3-OH butyrate was an important endometrial cancer biomarker even after adjusting for diabetes [49]. In the current study, 3-OH butyrate and its derivatives did not significantly discriminate between cases and controls after excluding women with type 2 diabetes mellitus. This may relate to the strong association between 3-OH butyrate and diabetes, with multiple studies suggesting that 3-OH butyrate is an early marker of insulin resistance, even in non-diabetic populations [51,52,53]. 3-OH butyrate has also been identified as a potential biomarker of low-grade female papillary thyroid cancer [54] and high-grade serous carcinoma of the ovary [55]. Knapp and colleagues found sphinganine, sphingosine, dihydroceramide and ceramide levels to be significantly elevated in endometrial cancer tissue compared to healthy endometrium [56]. Audet-Delage and colleagues reported sphingolipids to be significantly elevated in the serum of women with recurrent non-endometrioid endometrial cancer [39]. Sphingolipids are involved in inflammation, proliferation, cell migration and apoptosis [57]. Here, we found tricosanoyl and behenoyl sphingomyelins to be upregulated in the plasma of post-menopausal women with endometrial cancer. Further studies are needed to validate the utility of these biomarkers for endometrial cancer detection.

Metabolomic biomarkers that can identify aggressive endometrial cancer phenotypes are important for directing therapy. Here, several metabolites were shown to have potential for establishing tumour stage, the presence of LVSI and deep myometrial invasion (Figure 9 and Figure 10, respectively). Glycerophosphocholines, glycerophosphoethanolamines, heme and 3-OH butyrate were important predictors of LVSI while X-12847, X-17337, Homovanillate (HVA), X-23644, 3-OH butyrate and Tigloylglycine were important predictors of deep myometrial invasion. These results must be interpreted with caution given the small sample sizes. Heme, an iron-containing porphyrin, is an important source of electrons for electron transfer and has been shown to be elevated in the clinically aggressive type II endometrial cancer [39,58]. Homovanillate, a metabolite of dopamine, is a neurotransmitter originating from tyrosine [59]. We did not find any prior studies identifying HVA as a marker of deep myometrial invasion in endometrial cancer. These markers warrant validation in an independent cohort and their mechanistic links to endometrial cancer should be elucidated prior to clinical translation.

This study has several strengths. Our metabolomics methodology, using multiple approaches for metabolite separation and identification (Reverse Phase Liquid Chromatography and Hydrophilic Interaction Liquid Chromatography), helped maximise the number of metabolites identified. The use of artificial intelligence to select the best-performing metabolites and to qualify their performance in an independent sub-group of samples is a further strength, as this minimises the unwanted inflation of performance that occurs in the absence of independent testing. Identified metabolites showed sufficient accuracy for endometrial cancer detection (including early-stage tumours), especially when combined in a biomarker panel, and thus have good potential for clinical utility. Indeed, many of these metabolites have mechanistic links with the malignant transformation process. The use of obese controls maximises the chance that discriminatory metabolites are cancer-specific rather than obesity-related and sets our study apart from previous studies where apparently healthy controls (i.e., women with normal BMI) were used. 

A limitation of our study design is that our metabolite panel may not identify non-endometrioid-/non-obesity-related tumours. It is also unclear how well the biomarkers will perform in other high-risk groups such as the elderly, those with postmenopausal bleeding or Lynch syndrome. The relatively small sample size and the attendant difficulty in controlling for potential confounding factors is another limitation. Several discriminatory metabolites could not be biochemically identified, which limits their clinical implementation.

## 5. Conclusions

We found specific plasma metabolites to have potential for the detection of endometrial cancer in a cohort of women with class III obesity. A metabolomic signature based on the top ten performing metabolites showed good promise. Glycerophospholipids, specifically glycerophosphocholines and glycerophosphoethanolamines, were particularly important in differentiating endometrioid endometrial cancer from controls. These findings suggest that a simple blood-based test has the potential to enable the early detection of endometrial cancer and provides a basis for a minimally invasive screening tool for women with class III obesity. Further studies are needed to validate the biomarker candidates and elucidate their role in endometrial carcinogenesis. 

## Figures and Tables

**Table 1:** Clinicopathological characteristics of the cohort.

Participant Characteristics	Total Cohort (*n* = 136)	Cases (*n* = 67)	Controls (*n* = 69)	*p*-Value
Age (years) median(IQR)	54 (43,65)	63 (54,69)	46 (39,53)	<0.001
BMI (kg/m^2^) median (IQR)	46 (39,52)	40 (34,46)	50 (46,55)	<0.001
White ethnicity	121 (89.0%)	59 (88.1%)	62 (89.9%)	0.888
Ever smokers	50 (36.8%)	23 (34.3%)	27 (39.1%)	0.833
Nulliparity	48 (35.3%)	37 (55.2%)	11 (15.9%)	<0.001
Post-menopausal	77 (56.6%)	56 (83.6%)	21 (30.4%)	<0.001
History of diabetes mellitus	46 (33.8%)	17 (25.4%)	29 (42.0%)	0.04
**Tumour Characteristics**				
FIGO (2009)				
Grade 1	-	47 (70.2%)	-	
Grade 2	-	14 (20.9%)	-	
Grade 3	-	6 (9.0%)	-	
FIGO (2009)				
Stage 1	-	59 (88.0%)	-	
Stage 2	-	2 (3.0%)	-	
Stage 3	-	6 (9.0%)	-	
Myometrial invasion ≥50%	-	12 (18.0%)	-	
Presence of LVSI	-	12 (18.0%)	-	

**Table 2:** Random forest diagnostic accuracy based on the training set made from 80% cases and controls (*n* = 109, cancers = 54, controls = 55).

Actual Group	Predicted Group		
	Cancer	Control	Class Error
**Cancer**	48	6	0.11111
**Control**	9	46	0.16363

OOB Error rate: 13.76%. Number of Trees: 1000. Number of variables tried at each split: 33. Sensitivity: 88.9%, specificity: 83.6%.

**Table 3:** Random forest prediction accuracy applied on the testing set made from 20% of cases and controls (*n* = 27, cancers = 13, controls = 14).

Actual Group	Predicted Group		
	Cancer	Control	Class Error
**Cancer**	12	1	0.0769
**Control**	1	13	0.0714

OOB Error rate: 7.41%. Prediction accuracy: 92.6%. AUC: 0.95.

**Table 4:** Random forest diagnostic accuracy developed based on the training set made from 80% of stage 1 endometrial cancer cases and controls (*n* = 102, cancers = 47, controls = 55).

Actual Group	Predicted Group		
	Cancer	Control	Class Error
**Cancer**	41	6	0.1276
**Control**	9	46	0.16363

OOB Error rate: 14.71%. Number of Trees: 1000. Number of variables tried at each split: 22. Sensitivity: 87.2%, specificity: 83.6%.

**Table 5:** Random forest prediction accuracy applied on the testing set made from 20% of stage 1 endometrial cancer cases and controls (*n* = 26, cancers = 12, controls = 14).

Actual Group	Predicted Group		
	Cancer	Control	Class Error
**Cancer**	8	4	0.3333
**Control**	0	14	0.0000

OOB Error rate: 15.4%. Prediction accuracy: 84.6%.

**Table 6:** Pairwise correlation analysis for selected metabolites with age and BMI.

Correlation *p*−Value	Age	BMI	Parity	1-Lignoceroyl GPC	1-1 nyl-Steroyl-2-Linoleoyl-GPE	1-Linolenoyl GPC	1-1 Enyl-Steroyl GPE	3-OH-Butyryl Carnitine	1-1 Enyl Steroyl-2-Oleoyl GPE	3-OH Butyrate	X-24449	Eicosanodiote	1-2-Dilinoleoyl GPC
**Age**	1.0000												
**BMI**		1.0000											
**Parity**			1.0000										
**1-Lignoceroyl GPC**	0.4427 *	−0.5860 *	−0.2802 *	1.0000									
	0.0000	0.0000	0.0010										
**1-1 Enyl-Steroyl-2-Linoleoyl-GPE**	0.3953 *	−0.4879 *	−0.3287 *	0.7334 *	1.0000								
	0.0001	0.0000	0.0001	0.0000									
**1-Linolenoyl GPC**	0.3881 *	−0.4824 *	−0.2581 *	0.7474 *	0.6760 *	1.0000							
	0.0002	0.0000	0.0024	0.0000	0.0000								
**1-1Enyl-Steroyl GPE**	0.4518 *	−0.4846 *	−0.3270 *	0.6833 *	0.7022 *	0.6925 *	1.0000						
	0.0000	0.0000	0.0001	0.0000	0.0000	0.0000							
**3-OH-butyryl carnitine**	−0.2566	0.4438 *	0.2164 *	−0.6610 *	−0.7025 *	−0.6535 *	−0.4620 *	1.0000					
	0.1411	0.0000	0.0114	0.0000	0.0000	0.0000	0.0000						
**1-1EnylSteroyl-2-Oleoyl** **GPE**	0.4278 *	−0.4523 *	−0.2680 *	0.6379 *	0.8935 *	0.6511 *	0.7199 *	−0.5945 *	1.0000				
	0.0000	0.0000	0.0016	0.0000	0.0000	0.0000	0.0000	0.0000					
**3-OH butyrate**	−0.3204 *	0.3673 *	0.2196	−0.5891 *	−0.6927 *	−0.6334 *	−0.5204 *	0.8741 *	−0.6605 *	1.0000			
	0.0079	0.0000	0.0102	0.0000	0.0000	0.0000	0.0000	0.0000	0.0000				
**X-24449**	0.4580 *	−0.4386 *	−0.2566 *	0.6211 *	0.5864 *	0.4975 *	0.5026 *	−0.4870 *	0.4886 *	−0.4400 *	1.0000		
	0.0000	0.0000	0.0026	0.0000	0.0000	0.0000	0.0000	0.0000	0.0000	0.0000			
**Eicosanodiote**	0.3294 *	−0.3388 *	−0.2135 *	0.5632 *	0.6889 *	0.5576 *	0.5017 *	−0.5435 *	0.6143 *	−0.5169 *	0.4147 *	1.0000	
	0.0050	0.0000	0.0126	0.0000	0.0000	0.0000	0.0000	0.0000	0.0000	0.0000	0.0000		
**1-2-Dilinoleoyl GPC**	0.3001 *	−0.5158*	−0.3056 *	0.7042 *	0.7675 *	0.6640 *	0.5470 *	−0.6416 *	0.6019 *	−0.5968 *	0.5395 *	0.5786 *	1.0000
	0.0212	0.0000	0.0003	0.0000	0.0000	0.0000	0.0000	0.0000	0.0000	0.0000	0.0000	0.0000	

* p-value < 0.05. This was performed with pairwise correlation analysis with Bonferroni correction. There was no evidence of a strong correlation between age/BMI and selected metabolites.

**Table 7:** Random forest diagnostic accuracy developed based on the training set made from 80% of endometrial cancer cases and controls after exclusion of those with type 2 diabetes mellitus (*n* = 72, cancers = 40, controls = 32).

Actual Group	Predicted Group		
	Cancer	Control	Class Error
**Cancer**	38	2	0.0500
**Control**	6	26	0.1875

OOB Error rate: 11.11%. Number of Trees: 1000. Number of variables tried at each split: 73. Sensitivity = 95%, Specificity = 81%.

**Table 8:** Random forest prediction accuracy applied on the testing set made from 20% of endometrial cancer cases and controls after exclusion of women with type 2 diabetes mellitus (*n* = 18, cancers = 10, controls = 8).

Actual Group	Predicted Group		
	Cancer	Control	Class Error
**Cancer**	8	2	0.200
**Control**	0	8	0.0000

OOB Error rate 11.11%. Prediction accuracy 88.9%.

## Data Availability

Data are available through the corresponding author upon reasonable request.

## Supplementary Materials

The following are available online at https://www.mdpi.com/2072-6694/13/4/718/s1, Figure S1: Overview of study workflow, Figure S2: ROC curves based on Random Forest algorithms for the detection of endometrial cancer of all stages (a) and stage 1 endometrial cancer (b) using 80% of study samples and based on the top 10 discriminatory biomarkers, Table S1: Description of liquid chromatographic columns and mode of ionisation used in metabolite extraction based on protocols by Metabolon Inc, Table S2: Biochemical identities, super-pathways and sub-pathways of discriminatory metabolites for EC detection.
