# Supplementary material for: Metabolomic Biomarkers for the Detection of Obesity-Driven Endometrial Cancer
Source: Cancers (Basel). 2021 Feb 10;13(4):718. doi: 10.3390/cancers13040718 (PMC7916512; doi:10.3390/cancers13040718)
Supplement: Supplementary file 1 [file cancers-13-00718-s001.pdf]

Supplementary material

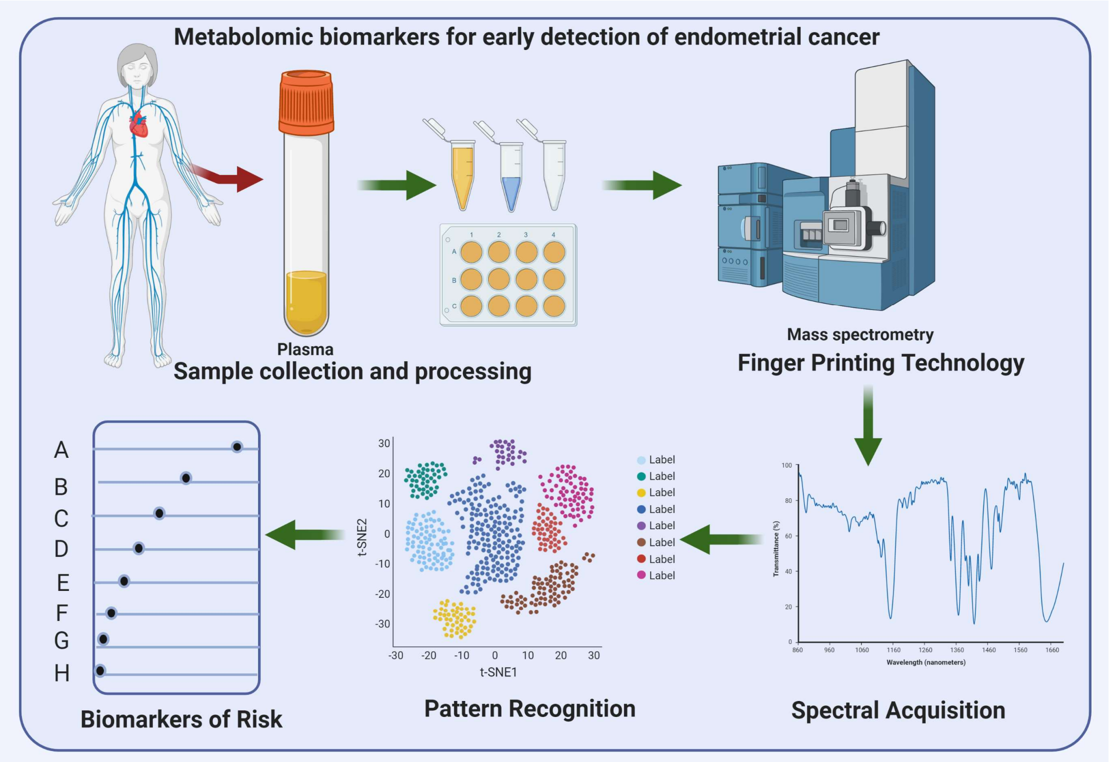

Figure S1: Overview of study workflow.

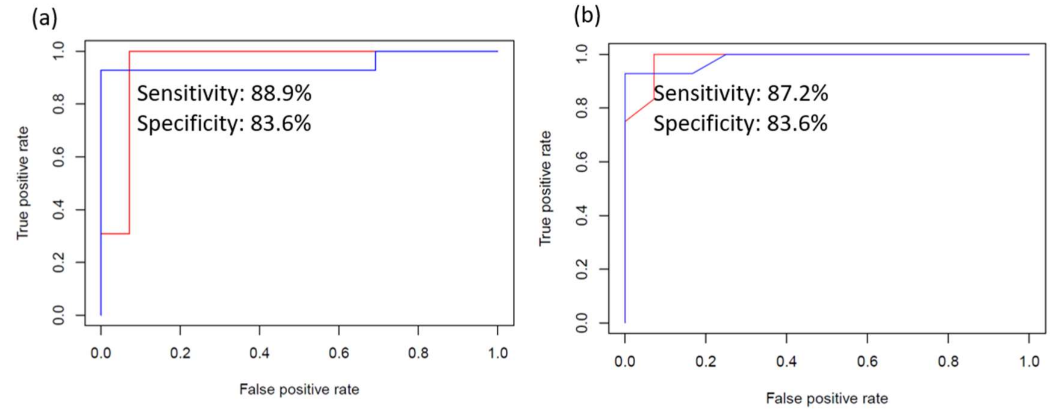

Figure S2: ROC curves based on Random Forest algorithms for the detection of endometrial cancer of all stages (a) and stage 1 endometrial cancer (b) using 80% of study samples and based on the top 10 discriminatory biomarkers

Table S1: Description of liquid chromatographic columns and mode of ionisation used in metabolite extraction based on protocols by Metabolon Inc.

Chromatographic technique	Nature of column	Constituents	Mode of ionisation
Reverse Phase Liquid Chromatography	C18 column (Waters UPLC BEH C18-2.1x100 mm, 1.7 μm)	Water and methanol, containing 0.05% perfluoropentanoic acid (PFPA) and 0.1% formic acid (FA).	Acidic positive ion conditions
		Methanol, acetonitrile, water, 0.05% PFPA and 0.01% FA	Acidic positive ion conditions
		Methanol, water and 6.5mM Ammonium Bicarbonate at pH 8.	Basic negative ion conditions
Hydrophilic Interaction Liquid Chromatography	HILIC column (Waters UPLC BEH Amide 2.1x150 mm, 1.7 μm)	Water and acetonitrile with 10mM Ammonium Formate, pH 10.8.	Basic negative ion conditions

Table S2: Biochemical identities, super-pathways and sub-pathways of discriminatory metabolites for EC detection

**Biochemical identity**	**Super pathway**	**Sub pathway**
1-(1-enyl-stearoyl)-2-linoleoyl-GPE (P-18:0/18:2)*	Lipid	Plasmalogen
1-(1-enyl-stearoyl)-2-oleoyl-GPE (P-18:0/18:1)	Lipid	Plasmalogen
1-(1-enyl-stearoyl)-GPE (P-18:0)*	Lipid	Lysoplasmalogen
1,2-dilinolenoyl-GPC (18:3/18:3)*	Lipid	Phospholipid Metabolism
1,2-dilinoleoyl-GPC (18:2/18:2)	Lipid	Phospholipid Metabolism
1,2-dilinoleoyl-GPC (18:2/18:2)	Lipid	Phospholipid Metabolism
1-lignoceroyl-GPC (24:0)	Lipid	Lysolipid
1-linolenoyl-GPC (18:3)*	Lipid	Lysolipid
1-oleoylglycerol (18:1)	Lipid	Monoacylglycerol
1-stearoyl-2-linoleoyl-GPC (18:0/18:2)*	Lipid	Phospholipid Metabolism
1-stearoyl-GPC (18:0)	Lipid	Lysolipid
3-hydroxybutyrate (BHBA)	Lipid	Ketone Bodies
3-hydroxybutyrylcarnitine	Lipid	Fatty Acid Metabolism(Acyl Carnitine)
Adipate	Lipid	Fatty Acid, Dicarboxylate
Arginine	Amino acid	Urea cycle; Arginine and Proline Metabolism
Azelate (nonanedioate)	Lipid	Fatty Acid, Dicarboxylate
Citrulline	Amino Acid	Urea cycle; Arginine and Proline Metabolism
Eicosanodioate	Lipid	Fatty Acid, Dicarboxylate
Sphingomyelin	Lipid	Sphingomyelin metabolism
Tryptophan	Amino acid	Tryptophan metabolism
